# Evaluation of cerebrospinal fluid penetration of delafloxacin in a murine model

**DOI:** 10.1128/aac.01660-25

**Published:** 2026-01-14

**Authors:** Marin Lahouati, Vasco Dias Meireles, Camille Rougnon-Glasson, Antoine Petitcollin, Fabien Xuereb

**Affiliations:** 1 Hôpital Pellegrin, Service de pharmacie clinique, CHU de Bordeaux, Bordeaux, France; 2 INSERM, BMC, U1034, Univ. Bordeaux, Pessac, France; 3 Laboratoire de Pharmaco-Toxicologie Biologique et Médico-Légale, CH Tarbes-Lourdes, Tarbes, France; Providence Portland Medical Center, Portland, Oregon, USA

**Keywords:** delafloxacin, pharmacokinetic, cerebrospinal fluid

## Abstract

Cerebrospinal fluid (CSF) penetration of delafloxacin was evaluated in a murine model. Mice received a single 40 mg/kg intraperitoneal dose. Plasma and CSF samples were collected at five time points over 4 h and analyzed by ultra high-performance liquid chromatography/tandem mass spectrometry (UPLC-MS/MS). Pharmacokinetic parameters, including CSF penetration ratio (AUC_0–4h_ CSF/AUC_0–4h_ plasma), were determined using a non-compartmental model. The CSF penetration ratio of delafloxacin was 49%. Cmax/MIC ratios met pharmacokinetic/pharmacodynamic targets for *Staphylococcus* and *Streptococcus* spp., but not Enterobacterales.

## INTRODUCTION

Delafloxacin is a fourth-generation fluoroquinolone available in Europe since 2019 (1). According to the literature, fluoroquinolones generally exhibit favorable penetration profiles into CSF (2). Pea et al. reported a cerebrospinal fluid (CSF)/plasma AUC ratio of 0.71 for levofloxacin in patients with external ventricular drains (3). For ciprofloxacin, CSF penetration ranges from 0.24 to 0.43 in non-inflammatory conditions and can reach up to 0.9 in the presence of meningeal inflammation (4). Moxifloxacin also demonstrates favorable CSF penetration, with CSF/plasma ratios ranging from 0.46 to 0.9 depending on the presence of inflammation (5, 6). The physicochemical properties of delafloxacin (low molecular weight, moderate lipophilicity), its broad antimicrobial spectrum, and enhanced activity in acid environments make it a promising candidate for the treatment of central nervous system (CNS) infections. However, no data are currently available regarding the penetration of delafloxacin into the CNS. The aim of this study is to evaluate the penetration of delafloxacin into the CSF of mice.

## METHODS

### Animals

Female C57BL/6J mice aged 12 weeks were used (*n* = 15). Euthanasia was performed by cervical dislocation.

### Chemicals

Delafloxacin was provided by Menarini (QUOFENIX). Delafloxacin powder was reconstituted with 0.9% NaCl and then diluted to obtain a solution with a concentration of 5 mg/L.

### CSF and blood sampling

Analgesia was obtained by subcutaneous injection of buprenorphine (0.05 mg/kg) 30 min before sampling. Mice were anesthetized with isoflurane (3% induction and 1% maintenance) (Virbac Schweiz, Glattbrugg, Germany) during blood and CSF sampling. For CSF sampling, the neck skin was incised, and muscles were dissected to expose the cisterna magna. CSF samples were collected from region 5 of the cerebellum/spinal cord using stereotaxis and a 1.3 mm glass capillary. CSF was diluted in 0.9% NaCl (1:10) and stored at −80°C in Eppendorf tubes. The retro-orbital route was used for blood sampling. Blood (500 µL) was transferred into Eppendorf tubes containing ethylene diamine tetra acetic acid (EDTA) as anticoagulant (1:10 v:v EDTA:blood). Plasma was separated by an 8-min centrifugation at 12,000 × *g* and then stored at −80°C in Eppendorf tubes.

### UPLC-MS/MS

Total delafloxacin concentration in plasma and CSF samples was assayed using ultra high-performance liquid chromatography/tandem mass spectrometry (UPLC-MS/MS).

### Pharmacokinetic study

The pharmacokinetic parameters of single-dose delafloxacin (40mg/kg) were evaluated. Delafloxacin was administered by intraperitoneal (IP) route. Mice (*n* = 15) were sampled 15 min, 30 min, 1 h, 2 h, and 4 h after delafloxacin administration (3 mice at each time point). Pharmacokinetic parameters (elimination constant [Ke], elimination half-life [t1/2], maximum concentration [Cmax], time to reach Cmax [Tmax], and area under the concentration-time curve from 0 to 4 h [AUC_0–4h_]) were calculated using a non-compartmental model with PK Solver software (version 2.0). The penetration ratio of the drug from the plasma into the CSF was calculated by the AUC_0–4h_ ratio (AUC_0–4h_ CSF/AUC_0–4h_ plasma). Pharmacokinetic parameters were evaluated based on total and unbound concentrations calculated with protein binding of 97.6% in plasma (7). Given the extremely low protein concentrations in the CSF, protein binding of delafloxacin within this compartment was assumed to be negligible.

### Pharmacodynamic/pharmacokinetic target

PK/PD target was defined as Cmax/MIC ratio > 12 in CSF based on literature reports, suggesting this ratio is associated with optimal bactericidal activity for fluoroquinolones (8). We use epidemiological cut-off MIC for delafloxacin as defined by European Committee on Antimicrobial Susceptibility Testing (EUCAST): 0.016 mg/L for *Staphylococcus* spp., 0.03 mg/L for *Streptococcus* spp., and 0.125 mg/L for Enterobacterales (9).

## RESULTS

**Fig 1:**
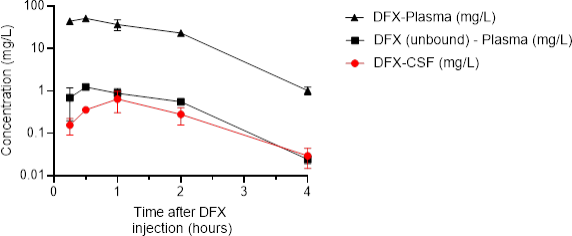
Plasma and cerebrospinal fluid (CSF) concentration-time profiles of delafloxacin (DFX) in mice received a single dose (40 mg/kg) of delafloxacin. Each symbol represents the mean value from the three animals. The red line represents CSF concentrations of DFX. The black line (triangles) represents total concentrations of DFX in plasma. The black line (squares) represents unbound concentrations of DFX in plasma.

Pharmacokinetic parameters are reported in Table 1. Mean maximal concentration in plasma (Cmax – total) was 51.51 mg/L (SD: 6.61 mg/L), and AUC (plasma-total)_0–4h_ was 93.72 mg/L*h. Mean unbound maximal concentrations (Cmax – plasma unbound) in plasma were 1.24 mg/L (SD: 0.16 mg/L), and AUC (plasma-unbound)_0–4h_ was 2.25 mg/L*h. CSF maximal concentration (Cmax – CSF) was 0.65 mg/L (SD: 0.34 mg/L), and AUC (CSF)_0–4h_ was 1.11 mg/L*h. AUC_0–4h_ penetration ratio of the drug from the plasma into the CSF was 1.2% for total concentration and 49.3% for unbound concentrations. Cmax/MIC ratios were 40.6 for *Staphylococcus* spp. and 21.7 for *Streptococcus* in CSF. Concerning Enterobacterales, Cmax/MIC was 5.2 in CSF. Concentration–time curves of delafloxacin in plasma and CSF are represented in Fig. 1.

**TABLE 1:** Pharmacokinetic parameters of delafloxacin^*a*^

Parameter	Plasma (total)	Plasma (unbound)	CSF
T_1/2_ (hours)	0.61		0.68
T_max_ (hours)	0.5		1
C_max_ (mg/L)	51.51	1.24	0.65
AUC_0–4h_ (mg/L*h)	93.72	2.25	1.11

^*a*^ A total of 15 animals were used, with three animals sampled per time point across five time points. CSF: cerebrospinal fluid; T_1/2_: elimination half-life, C_max_: maximum concentration; T_max_: time to reach C_max_; AUC: area under the concentration-time curve.

## DISCUSSION

These results provide the first data regarding the diffusion of delafloxacin into the cerebrospinal fluid (CSF) in mice. The CSF penetration ratio of delafloxacin in the absence of inflammation was 49%, which is comparable to values reported in the literature for other fluoroquinolones (2). Due to the high plasma protein binding of delafloxacin in mice (97.6%), the concentrations achieved in the CSF are limited. However, the PK/PD target of Cmax/MIC observed remains above 12 for *Staphylococcus* spp. and *Streptococcus* spp. In contrast, the Cmax/MIC ratio observed for Enterobacterales is lower (5.16), which is insufficient to achieve optimal bactericidal activity. This study has several limitations. First, interspecies variability, such as BBB characteristics and pharmacokinetics parameters, limits the direct extrapolation of our findings from mice to humans. For instance, delafloxacin plasma protein binding is higher in mice (97.6%) than in humans (83–84%), and elimination half-life is much shorter in mice (0.6 h vs. 12 h in humans) (1). These differences suggest that CSF penetration ratios observed in mice may not fully reflect delafloxacin penetration in humans; notably, the lower protein binding in humans could result in a higher CSF penetration (10). Moreover, this study was conducted under non-inflammatory conditions. Inflammation, as commonly observed during CNS infections (11), can significantly alter the permeability of BBB, potentially enhancing antibiotic penetration into the CSF (2), and, thus, additional studies are warranted to assess the impact of BBB disruption on delafloxacin penetration. We previously reported on a murine model simulating BBB disruption to study antibiotic pharmacokinetics under inflammatory conditions (12), which could be applied here to complement the current findings. Lastly, we measured delafloxacin penetration in the CSF, which may not reflect its penetration in the brain. It would, therefore, be relevant to assess brain penetration of delafloxacin using either brain tissue homogenates or cerebral microdialysis (2).

### Conclusion

The CSF penetration of delafloxacin appears to be comparable to that of other fluoroquinolones. While PK/PD targets may be achieved for *Staphylococcus* spp. and *Streptococcus* spp., they are unlikely to be reached for Enterobacterales primarily due to the high plasma protein binding of delafloxacin.
